# Association between event-related disclosure and posttraumatic growth: Targeting Japanese people, measuring attitudes toward disclosure, and examining event-related rumination as a mediating variable

**DOI:** 10.1371/journal.pone.0340671

**Published:** 2026-01-09

**Authors:** Yuto Kimura, Takahiro Kozuka

**Affiliations:** Department of Education and Human Development, Nagoya University, Nagoya, Japan; Jahangirnagar University, BANGLADESH

## Abstract

This study examined the association between event-related disclosure and posttraumatic growth (PTG). Specifically, it targeted the Japanese adult, assessed attitudes toward event-related disclosure, and examined event-related rumination as a mediating variable. A cross-sectional online survey was conducted among Japanese adults aged 20–59. Participants completed measures of demographic characteristics, stressful life events attributes, attitudes toward disclosure, event-related rumination, and PTG. Analysis of data from 480 individuals revealed that neither willingness to disclose nor resistance to disclose was directly associated with PTG. However, both willingness to disclose and resistance to disclose were positively associated with PTG through deliberate rumination and negatively associated with PTG through intrusive rumination. The effect sizes for willingness to disclose were approximately three times greater than those for resistance to disclose. These findings suggest that event-related disclosure may enhance PTG by promoting deliberate meaning-making processes, while also potentially hindering it by reinforcing involuntary negative thinking. Furthermore, these effects may be stronger when individuals are more proactive and disclose more frequently. These results have important implications for interventions aimed at enhancing PTG through event-related disclosure.

## Introduction

Experiencing stressful life events is common across the general population. According to a report by World Health Organization (WHO), the lifetime prevalence of experiencing a traumatic-level stressful life event is estimated at 70.4% globally [1]. It is assumed that most people have experienced at least one stressful event in their lifetime, including events not severe enough to cause trauma.

Stressful life events can affect individuals in various ways. For example, they may cause emotional disturbances such as anxiety and fear, unhealthy behaviors such as poor nutrition or excessive alcohol consumption, dysfunctions in the neuroendocrine system, and heightened autonomic nervous system arousal [2]. In some cases, people may develop posttraumatic stress reactions (PTSR), including intrusive thoughts, avoidance behaviors, negative changes in cognition and emotion, and heightened arousal and reactivity. If these symptoms persist for more than one month, individuals may be diagnosed with posttraumatic stress disorder (PTSD), which is classified as a mental disorder [3]. However, not all individuals develop maladaptive outcomes after experiencing stressful life events. Cohen et al. [2] reported that most individuals do not develop health problems following these experiences. Even in cases of highly stressful events capable of inducing trauma, the rate of PTSD diagnosis is 4.0% [1], which, although not negligible, is relatively low compared with the overall prevalence of event exposure. Moreover, most individuals with PTSD experience a reduction in the PTSR over time [4].

Thus, individuals can maintain psychological adaptation even after experiencing stressful life events. Among the various factors proposed to explain this adaptation, Tedeschi and Calhoun [5] introduced posttraumatic growth (PTG) as a psychological resource arising from such experiences. PTG is defined as “positive psychological change experienced as a result of a struggle with highly challenging life circumstances” [6]. Tedeschi and Calhoun [5] developed the Posttraumatic Growth Inventory (PTGI) to measure PTG. Since then, empirical studies employing the PTGI have accumulated evidence of an association between PTG and adaptive outcomes following stressful life events. Moreover, several meta-analyses have been synthesized this literature: Grace et al. [7] reviewed studies of brain injury patients, Sawyer et al. [8] synthesized studies of cancer or HIV/AIDS patients, Pięta and Rzeszutek [9] summarized studies of HIV patients, and Long et al. [10] reviewed studies across various event types. These studies have indicated that PTG is negatively associated with indicators of psychological distress, such as symptoms of depression and anxiety. They also demonstrated that PTG is positively associated with indicators of psychological adaptation, such as quality of life (QOL), well-being, and life satisfaction. Furthermore, these studies revealed positive associations between PTG and both subjective physical health and physical functioning.

Studies examining the association between PTG and PTSR or general stress responses suggest that PTG may help alleviate psychological distress. For instance, Chen et al. [11] provided longitudinal evidence that PTG is associated with reductions in PTSR. Matamela et al. [12] reported that PTG moderates the effect of traumatic events on PTSR. PTG can coexist with PTSR, and some studies suggest that PTG may arise as a response to PTSR. For instance, Shakespeare-Finch and Lurie-Beck [13] found a curvilinear (inverted U-shaped) association between PTSR and PTG, where PTG was lowest at both high and low levels of PTSR, and highest at moderate levels. Coroiu et al. [14] also demonstrated similar findings regarding the association between PTG and general stress. Furthermore, Dekel et al. [15] reported that the PTSR positively predicted PTG even ten years later in longitudinal data. To explain this process, how individuals struggle with psychological distress following stressful events, Tedeschi and Calhoun [6] proposed a theoretical model. This model has been revised and expanded based on empirical studies [16]. According to this model, demographic variables, personality traits, mental health status, stress exposure, and core beliefs shape individuals’ functioning prior to stressful events. After stressful events, the disruption of core beliefs triggers cognitive processing of the event, which can contributes to PTG. Interpersonal interactions, particularly event-related disclosure, also influence this cognitive processing. Ultimately, PTG leads to greater wisdom and the construction of life narratives, thereby fostering a more meaningful life trajectory [16].

Empirical studies of this model have focused on event-related rumination, a cognitive process that plays a central role in the PTG development. Event-related rumination refers to repetitive thoughts about a stressful life event and is categorized into two types: intrusive and deliberate rumination [5]. Intrusive rumination involves involuntary, often negative thoughts about the event; deliberate rumination refers to purposeful reflection on the event’s meaning or value [17]. The model posits that intrusive rumination emerges immediately after the events as a natural response but decreases over time. Conversely, deliberate rumination increases over time and facilitates the discovering meaning or value in events, thereby promoting PTG [5]. To assess these processes, Cann et al. [17] developed the Event-Related Rumination Inventory (ERRI), which has since been used in numerous empirical studies. A meta-analysis by Allen et al. [18] found that deliberate rumination consistently showed a positive association, whereas findings on intrusive rumination were inconsistent. While many studies reported a positive association, others reported a negative one. Romeo et al. [19] examined the association between intrusive rumination and PTG and found a negative correlation. They suggested that chronic intrusive rumination persisting long after an event may inhibit PTG.

Among interpersonal processes affecting such cognitive processing, event-related disclosure has received attention [6,16]. Event-related disclosure is defined as the sharing of thoughts and emotions related to stressful experiences with others [20]. According to the theoretical model [16], event-related disclosure may promote PTG by alleviating of negative emotions, conversing of involuntary thoughts into intentional reflections, and facilitating cognitive restructuring. These functions are also believed to promote PTG by reducing intrusive rumination and increasing deliberate rumination. Few studies have assessed whether people have disclosed their experiences (disclosure experience) and whether they wanted to do so (attitude toward disclosure) [20–22]. For example, Taku et al. [20] measured both with single item each and found that over 80% of participants who experienced stressful events had disclosed them, with approximately 80% disclosing willingly and approximately 20% disclosing unwillingly. They also found that people who disclosed exhibited higher PTG than those who did not disclose, regardless of their attitude.

Subsequently, several studies used validated multi-item scales to assess disclosure attitudes, such as the Distress Disclosure Index (DDI; Kahn & Hessling [23]), Social Constraints Scale (SCS; Lepore & Ituarte [24]), and the Disclosure of Trauma Questionnaire (DTQ; Müller et al. [25]). Among these, the DDI measures the tendency to disclose distress (positive attitude), whereas the SCS assesses perceived social constraints against disclosure (negative attitude). The DTQ evaluates both positive and negative disclosure attitudes and the perceived emotional expression during disclosure. However, PTG studies have typically used two DTQ subscales: positive and negative attitudes toward disclosure [26,27]. Thus, although the measurement and classification of disclosure attitudes have been clarified, their conceptual definitions and theoretical positioning remain vague in the literature. Therefore, this study first clarifies these points. We refer to two attitudes as “willingness to disclose” and “resistance to disclose.” According to Katz and Stotland [28], an attitude is a psychological tendency organized through experience that directs responses to a particular object. Based on this, attitude toward disclosure can be defined as a psychological state formed through past disclosure experiences that subsequently guides future disclosure behavior. Thus, willingness to disclose reflects an evaluative judgment that past disclosures were beneficial and encourages future disclosure, whereas resistance to disclose reflects the judgment that past disclosures were unhelpful or harmful and discourages future disclosure. Ryninks et al. [27] have found that willingness to disclose was positively correlated with disclosure frequency, whereas resistance to disclose showed no correlation. In other words, a high level of willingness to disclose reflects frequent disclosure, whereas resistance to disclose reflects infrequent disclosure.

Based on the theoretical model above, the following assumptions can be made about the association between attitudes toward disclosure and PTG: A high willingness to disclose likely indicates a current positive attitude, beneficial past experiences, and a high frequency of disclosure. In this case, event-related disclosure is likely to play a functional role in PTG development, resulting in less intrusive and more deliberate rumination, and ultimately higher PTG. In contrast, A high resistance to disclose likely reflects a current negative attitude, unhelpful or harmful past experiences, and low frequency of disclosure. In such cases, disclosure fails to fulfill its role in PTG development, so intrusive and deliberate rumination are unlikely to be affected, and PTG is not enhanced. Empirical studies to date generally support this theoretical background. That is, many studies reported a significant positive association between willingness to disclose and PTG, but no significant association between resistance to disclose and PTG [27,29,30].

Research on attitudes toward disclosure has gradually increased. However, he existing literature has several limitations. First, few studies have examined this association while considering rumination related to stressful events. As noted above, theoretical models suggest that disclosing an event may reduce intrusive rumination and increase deliberate rumination, thereby indirectly enhancing PTG. Therefore, it is necessary to investigate the association between disclosure attitudes and PTG with event-related rumination as a mediator. Most previous studies focused on specific types of stressful life events. However, Tedeschi et al. [16] suggested that PTG may develop through common pathways regardless of the event type. In research on event-related rumination, event type is typically not restricted, allowing for examination of the direct association between event-related rumination and PTG [17]. Similarly, research on event-related disclosure and PTG should avoid restricting the event type to identify findings generalizable across a range of events. Furthermore, most studies were conducted in Western cultural contexts. As PTG is influenced by cultural factors, it should also be examined in specific cultural contexts. Lindstrom et al. [22] suggested that the association between event-related disclosure and PTG may be more pronounced in Eastern cultures than in Western ones. Therefore, it is important to investigate this association within a clearly defined cultural setting, particularly Eastern cultures.

This study aimed to investigate the association between event-related disclosure and PTG in a Japanese sample, measuring attitudes toward disclosure with event-related rumination as a mediator. A wide variety of stressful events were considered, with event type treated as a control variable. This study carries important clinical implications. Previous studies examined interventions to promote PTG. Roepke [31] conducted a meta-analysis and reported that interventions, though modest in effect, could enhance PTG. One effective intervention identified was encouraging individuals to engage in event-related disclosure. However, despite evidence of its effectiveness, the study did not clarify which disclosure procedures are most effective, and this issue has received little attention in subsequent research. By clarifying the association between event-related disclosure and PTG, this study may contribute to developing interventions to enhance PTG.

The following hypotheses were tested in this study:

- Hypothesis 1: Willingness to disclose is positively associated with PTG through less intrusive and more deliberate rumination.
- Hypothesis 2: Resistance to disclose is unrelated to both types of event-related rumination and PTG.

## Methods

### Participants and procedure

An online survey was conducted from June 13–21, 2024, using a Japanese sample. The survey was created using Qualtrics (Qualtrics Inc.) and administered through Cross Marketing Inc. Participants first responded to the screening items, and those meeting the exclusion criteria were automatically excluded. Additional exclusion criteria were applied for the post hoc analysis. Ultimately, 480 of the 7,159 respondents were included in the analysis.

The exclusion criteria set for screening were as follows: First, regarding demographic variables, (a) participants under 20 or over 59 years of age were excluded. Participants were selected from age categories ranging from “19 and younger,” “20,” “21,”... “58,” “59,” and “60 and older.” Those who selected “19 and younger” or “60 and older” were automatically terminated. Next, regarding stressful life events and (b) participants whose event occurred more than 10 years ago were excluded. After recalling the most stressful life event experienced in the past, they chose either “within 10 years” or “more than 10 years ago.” Those who chose the latter option were excluded. In addition, participants who responded that the event occurred exactly “0 years and 1 month” ago were excluded post hoc. The reason for not excluding them initially was that this study was conducted in conjunction with an unpublished primary study, which did not exclude events occurring within one month. (c) Participants who rated the event as not stressful were excluded. Following Cann et al. [17], participants rated the stress level of the event identified in (b) on a 7-point Likert scale (1 = “not at all stressful” to 7 = “extremely stressful”). Those who selected 1–4 (up to “neither stressful nor stressful”) were automatically excluded. (d) Participants who never disclosed the event to anyone were excluded. Participants selected “Yes” or “No” to indicate whether they had ever disclosed the event identified in (b) and (c) to someone. Those who responded “No” were automatically excluded. (e) Participants who demonstrated insufficient cognitive engagement with the survey were excluded. An Instructional Manipulation Check (IMC) was used to identify participants who did not read the instructions [32]. The IMC item read as follows: “In internet-based surveys, it is sometimes problematic that some people lie, skip reading questions, or respond carelessly. Therefore, we would like to confirm whether you have read this sentence carefully. We sincerely apologize for the unusual request; however, if you read this sentence, please do not answer the following question (that is, do not select any of the options) and proceed to the next page.” Participants were presented with a 5-point Likert scale ranging from “Strongly disagree” to “Strongly agree.” Those who selected any option were excluded from the analysis. Additionally, a Directed Questions Scale (DQS) [33] was used to identify participants who did not pay adequate attention to the Likert-scale items. These items included instructions such as, “For this item, please select the option on the far left (right).” Participants who failed to select the instructed response were excluded from the analysis after completing the survey.

The present study did not screen based on whether the event qualified as trauma or met the PTSD diagnostic criteria. Posttraumatic growth (PTG) is predicted on the assumption that stressful life events challenge core beliefs, and it does not necessarily require the occurrence of trauma [16]. Moreover, several studies have indicated that PTG can arise not only from severe life events such as trauma but also from everyday stressful events [14,34]. Therefore, the present study identified target stressful life events based on the following criteria: (1) the event was perceived by participants as the most stressful event they had experienced in their lives, and (2) the event was genuinely experienced as stressful.

### Measures

#### Demographic variables.

The participants were asked to report their age and gender. Age was categorized as described previously. Gender options included “Male,” “Female,” and “Other.”

#### Attributes of stressful life events.

Participants were asked about the type of event, the time elapsed since the event, and the person to whom they had disclosed the event. Regarding the time elapsed from the event, we asked participants to answer using the method described in the screening section. The types of events were categorized based on prior Japanese studies [35,36] including “Relationship” (e.g., romantic breakup, bullying, online harassment), “Main job” (e.g., exam failure, job loss, harassment), “Self” (e.g., personal illness, accidents, victimization), “Family” (e.g., parental divorce, abuse, family illness) and “Bereavement” (e.g., death of close family or friends). Furthermore, participants who did not fit into any category were instructed to select “Other.” This study differs from previous studies [35,36] in that it included not only university students but also employed adults. Therefore, we established the “Main job” category, which included job search failures and layoffs; these would otherwise fall under “academics” for students. Regarding the person whom they disclosed the event, participants were asked to choose from “Family” (e.g., parent, partner, or sibling), “Friend” (e.g., close friend or peer), and “Expert” (e.g., doctor, counselor, or social worker); participants whose response did not fit into any category were instructed to select “Other.” For both event type and recipient of disclosure, participants who selected “Other” were asked to provide a free-text description. In the analysis, these free-text responses were reviewed, and if they matched an existing category, they were recategorized accordingly. If no suitable category existed, the response remained classified as “Other.”

#### Attitudes toward event-related disclosure.

To measure attitudes toward event-related disclosure, we used the Japanese scale developed by Kimura and Kozuka [37], based on the DTQ developed by Müller et al. [25]. This scale measures attitudes toward disclosure associated with stressful life events. It consists of two subscales, each with four items: willingness to disclose and resistance to disclose. Although Kimura and Kozuka [37] confirmed the reliability and validity of this scale, it was used with minor modifications. Katz and Stotland [28] proposed the following three components of attitudes: cognitive elements, which involve the recognition of an object’s attributes; evaluative elements, which involve forming judgments based on multiple cognitive elements; and behavioral elements, which involve forming behavioral intentions based on evaluative elements. Although the scale developed by Kimura and Kozuka [37] can assess attitudes toward event-related disclosure, willingness to disclose factor primarily consists of items that represent evaluative elements, whereas resistance to disclose is mainly composed of items that reflect behavioral elements. An imbalance in the representation of these factors may complicate the interpretation of the results. Katz and Stotland [28] indicated that evaluative elements are the fundamental components of attitudes. Therefore, we have revised the items in the resistance to disclose subscale to reflect the evaluative elements of attitude. Revisions to the item expressions are provided in the S1 File. Items were scored on a 6-point Likert scale ranging from 1 (*Not at all*) to 6 (*Completely applicable*). Confirmatory factor analysis demonstrated an acceptable model fit: Comparative Fit Index (CFI) =.96, Root Mean Square Error of Approximation (RMSEA) =.10, and Standardized Root Mean Square Residual (SRMR) =.08, indicating that the scale had a reasonable degree of validity. Cronbach’s alpha and McDonald’s omega coefficients for willingness to disclose were *α* = .89 and *ω* = .89, and for resistance to disclose were *α* = .82 and *ω* = .83, indicating sufficient reliability.

#### Event-related rumination.

To measure event-related rumination, we used the Japanese version of the Event-Related Rumination Inventory (ERRI-J), originally developed by Cann et al. [17] and adapted for use in the Japanese population by Taku et al. [38]. This scale measures the repetitive thoughts associated with stressful life events. Taku et al. [38] confirmed its reliability and validity. It comprises two factors, each 10 items: intrusive rumination and deliberate rumination. Items were scored on a 4-point Likert scale ranging from 1 (*Not at all*) to 4 (*Often*). In this study, Cronbach’s alphas were.97 for intrusive rumination and.96 for deliberate rumination. McDonald’s omegas were.97 for intrusive rumination and.96 for deliberate rumination. These results indicate sufficient reliability.

#### Posttraumatic growth.

To measure PTG, we used the short Japanese version of the Post-Traumatic Growth Inventory-X (PTGI-X-J-SF). This scale was translated into Japanese and shortened by Oshiro et al. [39] from the original scale developed by Tedeschi et al. [40]. It assesses positive psychological changes resulting from struggling with stressful life events. Oshiro et al. [39] confirmed the reliability and validity of the scale. This scale comprised a single factor. Items were scored on a five-point Likert scale ranging from 1 (*Not experienced at all*) to 5 (*Experienced a great deal*). In this study, Cronbach’s alpha was.91 and McDonald’s omega was.91, indicating sufficient reliability.

### Ethics considerations

This study was approved by the Ethics Review Board of the Graduate School of Education and Human Development at Nagoya University (Approval Number: 24–2163). Before starting the online survey, the following guidelines were displayed on the screen, and written informed consent was obtained electronically: participants were informed that they would be asked about past stressful experiences but were not required to provide answers about particularly distressing events unless they felt comfortable doing so; participation in the survey was voluntary and participants could freely choose not to participate; they could withdraw their consent and stop answering even after agreeing to participate; their privacy would be protected; and survey participation could evoke emotional distress. Contact information from the researchers and referral information for mental health support services were provided to participants who experienced distress during the survey. Participants were instructed to read these guidelines carefully, after which they were asked whether they consented to participate by selecting either “Yes” or “No.” Those who consented selected “Yes” and proceeded to the subsequent questions. In contrast, participants who did not consent selected “No,” and their responses were automatically terminated at that point. After completing the questionnaire, the participants were asked for permission to use their responses for the analysis. Responses from participants who did not provide permission were excluded from the analysis. Through this procedure, we confirmed that all the participants whose responses were included in the analysis explicitly consented to the use of their data.

### Data analysis

All analyses were conducted using R [41]. A confirmatory factor analysis was performed using the Lavaan package. Model fit was assessed based on the criteria of Hu and Bentler [42]: CFI ≥ .95, RMSEA ≤ .06, and SRMR ≤ .08 indicated a good fit; CFI = .90–.94, RMSEA = .07–.10, and SRMR = .09–.10 indicated an acceptable fit. Cronbach’s alpha and McDonald’s omega coefficients were used to determine scale reliability. Pearson’s correlation coefficients were calculated to examine the correlations between the factors. Structural equation modeling (SEM) was used to analyze the association between attitudes toward event-related disclosure, event-related rumination, and PTG. The model fit criteria were the same as those used for confirmatory factor analysis. A mediation analysis using a bootstrap method with 5,000 resamples was conducted to examine the indirect effects of event-related disclosure on PTG mediated by event-related rumination. A significant difference was deemed to exist when the 95% confidence interval excluded zero.

## Results

### Descriptive statistics

The dataset analyzed in this study is provided in the S2 File. Table 1 shows the characteristics of the participants and the attributes of the stressful life events. Specifically, the participants included 244 men, 234 women, and 2 others. The mean age was 48.36 years (*SD* = 8.64); 264 participants were in their fifties, 139 in their forties, 57 in their thirties, and 20 in their twenties. Regarding event type, 168 participants categorized their experience as “Main job,” 110 as “Family,” 99 as “Self,” 70 as “Relationships,” and 32 as “Bereavement,” while 1 participant responded with “Other.” The mean time elapsed since the event was 4.65 years (SD = 3.08). Additionally, 68 participants reported that the time elapsed since the event as 9–10 years, 62 reported 0–1 year (excluding one month), 57 reported 2–3 years, 57 reported 5–6 years, 54 reported 1–2 years, 49 reported 3–4 years, 38 reported 4–5 years, 33 reported 8–9 years, and 32 reported 7–8 years. Regarding the recipients of disclosure, 269 participants chose “Family,” 134 chose “Friend,” 58 chose “Expert,” and 19 chose “Other.”

**Table 1:** Demographic variables and the attributes of the stressful life event.

Variable		*N* = 480	%
Sex			
1	Man	244	50.83
2	Woman	234	48.75
3	Other	2	0.42
Age (M = 48.36, SD = 8.64)			
1	20 - 30	20	4.17
2	30 - 40	57	11.88
3	40 - 50	139	28.96
4	50 - 60	264	55.00
Event type			
1	Self	99	20.63
2	Relationship	70	14.58
3	Main job	168	35.00
4	Family	110	22.92
5	Bereavement	32	6.67
6	Other	1	0.21
Time from event (M = 4.65, SD = 3.08)			
1	0 - 1	62	12.92
2	1 - 2	54	11.25
3	2 - 3	57	11.88
4	3 - 4	49	10.21
5	4 - 5	38	7.92
6	5 - 6	57	11.88
7	6 - 7	30	6.25
8	7 - 8	32	6.67
9	8 - 9	33	6.88
10	9 - 10	68	14.17
Recipient of disclosure			
1	Family	269	56.04
2	Friend	134	27.92
3	Expert	58	12.08
4	Other	19	3.96

### Structural equation modeling analysis

Table 2 shows the means, standard deviations, Cronbach’s alpha values, and correlations among all scales. Pearson’s correlation coefficients indicated that willingness to disclose positively correlated with intrusive rumination (*r* = .42, *p* < .01), deliberate rumination (*r* = .43, *p* < .01), and PTG (*r* = .20, *p* < .01). In contrast, resistance to disclose was not correlated with intrusive rumination (*r* = .04, *n.s.*), deliberate rumination (*r* = .02, *n.s.*), or PTG (*r* = −.03, *n.s.*). Furthermore, a significant negative correlation was observed between willingness to disclose and resistance to disclose (*r* = −.21, *p* < .01). SEM was conducted to investigate the association between attitudes toward event-related disclosure, event-related rumination, and PTG (see Table 3). The model showed an excellent fit: CFI = 1.00, RMSEA = .00, and SRMR = .00, indicating that willingness to disclose had a positive association with PTG (*β* = .11, *p* < .05), intrusive rumination (*β* = .42, *p* < .01), and deliberate rumination (*β* = .43, *p* < .01). Meanwhile, resistance to disclose was not associated with PTG, but showed a positive association with intrusive rumination (*β* = .15, *p* < .01) and deliberate rumination (*β* = .13, *p* < .01). Furthermore, intrusive rumination was negatively associated with PTG (*β* = −.40, *p* < .01), while deliberate rumination was positively associated with PTG (*β* = .63, *p *< .01).

**Table 2:** Descriptive statistics, reliability, and correlation coefficient among variables.

Variable	*M*	*SD*	*α*	*ω*	1	2	3	4
Attitudes toward event-related disclosure								
Willingness to disclose (1)	10.90	5.07	.89	.89	–			
Resistance to disclose (2)	11.25	4.62	.82	.83	−.21**	–		
Event-related rumination								
Intrusive rumination (3)	24.78	9.06	.97	.97	.42**	.04 *n.s.*	–	
Deliberate rumination (4)	22.50	8.61	.96	.96	.43**	.02 *n.s.*	.71**	–
Posttraumatic growth (5)	26.63	12.88	.91	.91	.20**	−.03 *n.s.*	.07 *n.s.*	.38**

* *p* < .05

** *p* < .01

**Table 3:** Structural equation modeling analysis results.

Independent variable	Dependent variable	*β*	*SE*	*p*	95%CI	
Willingness to disclose	Intrusive rumination	.42	.04	.00	.35	.50
	Deliberate rumination	.43	.04	.00	.35	.50
	PTG	.11	.05	.01	.03	.20
Resistance to disclose	Intrusive rumination	.15	.04	.00	.07	.24
	Deliberate rumination	.13	.04	.00	.05	.21
	PTG	–	–	–	–	–
Intrusive rumination	PTG	−.40	.06	.00	−.51	−.29
Deliberate rumination		.63	.05	.00	.52	.73

### Mediation analysis by bootstrap method

The indirect effects were examined via mediation analysis using the bootstrap method (see Table 4). As a result, willingness to disclose had no direct positive association with PTG (*β* = .10, 95% CI [−.01,.55]), but showed an indirect negative association mediated by intrusive rumination (*β* = −.15, 95% CI [−.59, −.28]), and an indirect positive association mediated by deliberate rumination (*β* = .24, 95% CI [.50,.90]). By contrast, resistance to disclose also had no direct association with PTG (*β* = −.01, 95% CI [−.30,.23]), but showed an indirect negative association mediated by intrusive rumination (*β* = −.06, 95% CI [−.30, −.06]), and an indirect positive association mediated by deliberate rumination (*β* = .07, 95% CI [.07,.40]).

**Table 4:** Mediation analysis of the indirect effect of disclosure attitude on PTG through event-related rumination.

Independent variable	Mediating variable	Dependent variable	*β*	*SE*	95%CI	
Willingness to disclose	= = = = = =>	PTG	.10	.14	−.01	.55
	= > Intrusive rumination =>		−.15	.08	−.59	−.28
	= > Deliberate rumination =>		.24	.10	.50	.90
Resistance to disclose	= = = = = =>		−.01	.14	−.30	.23
	= > Intrusive rumination =>		−.06	.06	−.30	−.06
	= > Deliberate rumination =>		.07	.09	.07	.40

These findings indicate that neither willingness nor resistance to disclose is directly associated with PTG. Instead, both attitudes showed an indirect association through event-related rumination. Specifically, both attitudes were negatively associated with PTG through intrusive rumination and positively associated with deliberate rumination. However, the indirect effect sizes for willingness to disclose are approximately three times larger than those for resistance to disclose.

## Discussion

This study examined the association between event-related disclosure and PTG. Specifically, this study targeted Japanese participants, measured event-related disclosure in terms of attitudes toward disclosure, and included event-related rumination as the mediating variable. We examined the following hypothesis: (Hypothesis 1) willingness to disclose is positively associated with PTG, mediated by both intrusive rumination and deliberate rumination; and (Hypothesis 2) resistance to disclose is not associated with either type of rumination or with PTG. The results indicated that neither willingness to disclose nor resistance to disclose was directly associated with PTG. However, both variables demonstrated indirect associations mediated by event-related rumination: a negative indirect association with PTG via intrusive rumination and a positive indirect association with PTG via deliberate rumination. Moreover, the effect size of the association between willingness to disclose and PTG was approximately three times larger than that of resistance to disclose.

These findings indicate that disclosure attitudes are not directly associated with PTG, and that these associations are entirely mediated by event-related rumination. This suggests that previously reported direct associations between disclosure attitudes and PTG [27,29,30] may, in fact, have reflected indirect associations mediated by rumination. While the implications of these indirect associations are discussed in more detail below, we first consider why previous studies may have reported direct associations, giving the effect sizes found in this study. The indirect positive association between willingness to disclose and PTG via deliberate rumination was *β* = .24, while the indirect negative association via intrusive rumination was *β* = −.15. The sum of these effects (*β* = .09) suggests a small but meaningful overall association, which may have been interpreted as a direct association in earlier studies. In contrast, the indirect positive association between resistance to disclose and PTG through deliberate rumination was *β* = .07, and a negative indirect association via intrusive rumination was *β* = −.06, resulting in a negligible total effect (*β* = .01), generally considered too small to be meaningful. This may explain why prior studies have often found no direct association between resistance to disclose and PTG.

We now discuss the indirect associations between attitudes toward disclosure and PTG. The results showed that willingness to disclose was indirectly and positively associated with PTG through deliberate rumination, partially supporting Hypothesis 1 based on the theoretical models proposed by Tedeschi and Calhoun [6] and Tedeschi et al. [16]. This suggests that when people perceive disclosing stressful life events as beneficial and are willing to disclose them, they tend to disclose them frequently. This facilitates the functional role of disclosure, such as alleviating negative emotions, transforming involuntary reactions into intentional reflection, and promoting the cognitive processing of experiences. These functions, in turn, enhance deliberate rumination, a reflective, meaning-exploration cognitive process that contributes to PTG. Conversely, Hypothesis 1 also posited that willingness to disclose would be positively associated with PTG through the reduction of intrusive rumination, under the assumption that frequent disclosure would fulfill the functional role of disclosure and decrease intrusive rumination. However, the results showed that willingness to disclose was indirectly associated with lower PTG through increased intrusive rumination, contradicting this hypothesis. This is consistent with findings from research on psychological debriefing, which have shown that encouraging disclosure after stressful events can sometimes increase the intrusive recall of the event. Such increases in intrusive cognition may result from disorganized memory encoding or secondary traumatization triggered by the act of disclosure [43]. Taken together, these findings suggest that frequent event-related disclosure may, in some cases, increase intrusive rumination and hinder PTG.

Previous studies have reported that resistance to disclose is uncorrelated with disclosure frequency [27]. Based on this, we hypothesized (Hypothesis 2) that when resistance to disclose is high and disclosure frequency is low, event-related disclosure would not fulfill its role and thus would have no association with rumination or PTG. However, the results of this study revealed that resistance to disclose, such as willingness to disclose, was indirectly negatively associated with PTG via intrusive rumination and positively associated with deliberate rumination, thus providing no support for Hypothesis 2. First, the participants included in the present analysis disclosed their experiences at least once. Taku et al. [20], in a study that examined both whether participants had disclosed and whether they wanted to disclose, found that PTG was higher among individuals who had disclosed, regardless of their willingness to do so. This suggests that the impact of disclosure on rumination and PTG may depend not on attitudes or frequency alone but on whether disclosure occurred first.

Notably, the effect size of the indirect association between willingness to disclose and PTG was approximately three times larger than that of reluctance to disclose. This finding suggests that a positive attitude toward disclosure and frequent disclosure may amplify its influence of disclosure on rumination and PTG. By contrast, a negative attitude and infrequent disclosure may minimize such effects. These findings have not been addressed in previous studies and therefore represent a novel contribution of the present research. However, the current findings do not clarify the distinction between pathways that enhance PTG and those that hinder it, particularly in relation to event-related disclosure. Further research is required to examine this issue in detail. For example, Wadey et al. [44] suggest that event-related disclosure may be categorized into several types depending on the perspective from which people disclose their stressful life events. Therefore, it may be useful to investigate the nature of disclosure in greater detail, specifically the aspects of stressful life events that people choose to disclose. Additionally, previous studies have suggested that the recipients’ responses to disclosure may play an important role in fostering PTG [20]. Thus, future research should explore the interpersonal dynamics of event-related disclosure, focusing on the interactions between the discloser and the recipient.

## Limitations

This study had several limitations. First, because this study employed a cross-sectional design, it was not possible to determine whether event-related disclosure causally promotes posttraumatic growth (PTG) through event-related rumination mediation. Future research should adopt longitudinal designs to clarify the directionality of these associations. Second, all variables were measured using self-report instruments in this study. Previous studies have noted the potential influence of cognitive biases on self-report scores, especially for PTG [45]. To enhance the validity of the findings, future studies should incorporate alternative assessment methods such as behavioral indicators or informant reports. Third, while a wide range of stressful life events was included as control variables to examine the general mechanisms underlying PTG, this approach may limit the clinical applicability of the findings. For practical use in intervention settings, future research should narrow the scope to specific event types, examine how the variables relate within these contexts, and identify effective intervention strategies. Finally, the study focused on Japanese people, who belong to an East Asian cultural context in which event-related disclosure may function differently than in Western cultures. This sampling decision was based on prior findings suggesting that event-related disclosure plays a more significant role in PTG in East Asian populations [22]. However, to assess the cultural generalizability of the results, future studies should replicate this research across other East Asian and Western populations.

## Conclusion

This study examined the association between disclosure attitudes and posttraumatic growth (PTG), with event-related rumination as a mediating variable. The results showed that neither willingness to disclose nor reluctance to disclose was directly associated with PTG. However, both variables exhibited an indirect negative association with PTG through intrusive rumination and an indirect positive association through deliberate rumination. In addition, the association between willingness to disclose and PTG had an effect size approximately three times larger than that between reluctance to disclose and PTG. Considering the clinical implications of these findings, encouraging individuals who experience persistent posttraumatic stress reactions (PTSR), impaired health, or maladjustment following stressful life events to disclose their experiences during counseling or psychotherapy may promote deliberate rumination and consequently increase PTG, potentially leading to greater psychological and physical adaptation. However, repeatedly encouraging individuals to disclose their experiences may heighten intrusive rumination, thereby hindering PTG and disrupting psychological adjustment. Therefore, when implementing such interventions, it may be beneficial to encourage disclosure in a way that avoids focusing on the negative aspects of the event and instead emphasizes efforts to find meaning and value in the experience, which may promote PTG through deliberate rumination. Moreover, before and after interventions, it may be important to assess the cognitive state of the individual through dialogue, standardized scales, or other methods to appropriately tailor the intervention to their current state of mind.

## Supporting information

S1 File Specific revisions to item wording. (DOC)

S2 File Anonymized data set. (CSV)
