# Supplementary material for: Association between event-related disclosure and posttraumatic growth: Targeting Japanese people, measuring attitudes toward disclosure, and examining event-related rumination as a mediating variable
Source: PLoS One. 2026 Jan 9;21(1):e0340671. doi: 10.1371/journal.pone.0340671 (PMC12788629; doi:10.1371/journal.pone.0340671)
Supplement: Supplementary file 1 [file pone.0340671.s001.doc]

Before revise
その出来事について頭で考えることはよくあるが，誰かに話すことはあまりない。
(I often think about the event, but do not talk about it very much.)
その出来事で自分が経験したことについて話すタイミングがわからない。
(I never find the right time to talk about what I experienced during the event.)
その出来事について，誰かにありのままに話したことはない。
(I have not told anyone exactly what happened during the event.)
その出来事については，まだ誰にも話していない。
(I have not told anybody about the event.)
**After revise**
その出来事について頭で考えることはよくあるが，誰かに話そうとは思わない。
(I often think about the event, but don't want to talk about it very much.)
その出来事で自分が経験したことについて話すタイミングがわからない。
(I never find the right time to talk about what I experienced during the event.) = not revised
その出来事について，誰かにありのままに話す必要はない。
(I need not tell anyone exactly what happened during the event.)
その出来事については，誰にも話そうと思わない。
(I don't want to told anybody about the event.)
